# Estimation of genetic parameters and detection of chromosomal regions affecting the major milk proteins and their post translational modifications in Danish Holstein and Danish Jersey cattle

**DOI:** 10.1186/s12863-016-0421-2

**Published:** 2016-08-02

**Authors:** Bart Buitenhuis, Nina A. Poulsen, Grum Gebreyesus, Lotte B. Larsen

**Affiliations:** 1 Department of Molecular Biology and Genetics, Center for Quantitative Genetics and Genomics, Aarhus University, Blichers Allé 20, P.O. Box 50, Tjele, DK-8830 Denmark; 2 Department of Food Science, Aarhus University, Blichers Allé 20, P.O. Box 50, Tjele, DK-8830 Denmark

**Keywords:** Genetic parameters, Genome-wide association, Casein, Whey, Post-translational modification

## Abstract

**Background:**

In the Western world bovine milk products are an important protein source in human diet. The major proteins in bovine milk are the four caseins (CN), α_S1_-, α_S2_-, β-, and k-CN and the two whey proteins, β-LG and α-LA. It has been shown that both the amount of specific CN and their isoforms including post-translational modifications (PTM) influence technological properties of milk. Therefore, the aim of this study was to 1) estimate genetic parameters for individual proteins in Danish Holstein (DH) (*n* = 371) and Danish Jersey (DJ) (*n* = 321) milk, and 2) detect genomic regions associated with specific milk protein and their different PTM forms using a genome-wide association study (GWAS) approach.

**Results:**

For DH, high heritability estimates were found for protein percentage (0.47), casein percentage (0.43), k-CN (0.77), β-LG (0.58), and α-LA (0.40). For DJ, high heritability estimates were found for protein percentage (0.70), casein percentage (0.52), and α-LA (0.44). The heritability for G-k-CN, U-k-CN and GD was higher in the DH compared to the DJ, whereas the heritability for the PD of α_S1_-CN was lower in DH compared to DJ, whereas the PD for α_S2_-CN was higher in DH compared to DJ. The GWAS results for the main milk proteins were in line what has been earlier published. However, we showed that there were SNPs specifically regulating G-k-CN in DH. Some of these SNPs were assigned to casein protein kinase genes (*CSNK1G3, PRKCQ*).

**Conclusion:**

The genetic analysis of the major milk proteins and their PTM forms revealed that these were heritable in both DH and DJ. In DH, genomic regions specific for glycosylation of k-CN were detected. Furthermore, genomic regions for the major milk proteins confirmed the regions on BTA6 (casein cluster), BTA11 (*PEAP*), and BTA14 (*DGAT1*) as important regions influencing protein composition in milk. The results from this study provide confidence that it is possible to breed for specific milk protein including the different PTM forms.

**Electronic supplementary material:**

The online version of this article (doi:10.1186/s12863-016-0421-2) contains supplementary material, which is available to authorized users.

## Background

In the Western world bovine milk products are an important protein source in human diet. The major proteins in bovine milk are the four casein (CN), α_S1_-, α_S2_-, β-, and k-CN which occur in the approximate ratio of 4:1:4:1 (w/w) in milk, and the two whey proteins, β-lactoglobulin (β-LG) and α-lactalbumin (α-LA), which occur in the mutual ratio of 3:1 in milk (w/w) [1–3]. Total protein yield is an important part of the dairy milk payment system and has therefore been included in the dairy cattle breeding goal [4]. Different genetic variants of the CN genes have an influence on the amount of CN in the milk, as well as on the cheese making properties of milk [5–7]. It has been shown that in poorly coagulating milk samples of the Danish Holstein (DH) and Danish Jersey (DJ) breeds, the predominant combination of genotypes was BB for α_S1_-CN, A^2^A^2^ for β-CN, and AA for k-CN [8]. More recently, it has been shown that both the amount of specific CN and their post-translational modifications (PTM) have profound influence on the milk coagulation properties [6, 9]. Thus, breeding for detailed milk protein composition has attracted increased attention.

Apart from the disulphide bonds in the α_S2_-CN dimer and the k-CN multimer, PTMs of the bovine caseins include phosphorylation of α_S1_-CN, α_S2_-CN, β-CN and k-CN, as well as glycosylation also of k-CN [2, 6, 10]. Phosphorylation of α_S1_-CN results in a major form with eight phosphorylated serine residues (8P-α_S1_-CN) and a minor form with nine phosphorylated serine residues (9P-α_S1_-CN). For α_S2_-CN, the major phosphorylated form contains 11 phosphate groups (11P-α_S2_-CN) and the minor form 12 phosphate groups, while β-CN is usually present with five phosphate residues and k-CN with one residue [11]. The glycosylation degree varies with approximately 30-60 % of k-CN bring glycosylated, while 95 % is phosphorylated with 1-3 phosphate groups [9, 12]. Although there are different forms of this bovine protein due to multilevel phosphorylation and glycosylation, the mono-phosphorylated non-glycosylated k-CN is the predominant (>50 %) [12].

CN proteins in the milk can form a multi-molecular structure called the CN micelle, which plays an important role in the coagulation of milk. The PTMs of the CN influence both stability and size of the CN micelles [13, 14] and thereby influences technological properties of milk [6, 13, 15].

The majority of the studies reporting genetic variation for milk protein content are based on protein percentage or protein yield (e.g. [16–18]). These studies show that there is substantial genetic variation for total protein in bovine milk. Though only relatively few studies have reported genetic parameters for the detailed milk composition [19–21]. The individual CN and whey proteins show genetic variations in the coding sequences resulting in structural genetic variants [5]. These genetic variants have different expression levels, presumably due to further mutations in the regulating elements leading to differential expression levels [5]. Furthermore, an association study for detailed protein composition showed that the main regions associated with protein percentage and protein composition were located on chromosomes 5, 6, 11, and 14 [22]. Recently it was shown that the PTM of milk protein, like glycosylation of k-CN, shows genetic variation [15]. Furthermore Bijl et al. [23] showed that α_S1_-CN isoforms representing 8 or 9 phosphorylations, respectively, showed genetic variation and apparently was regulated by different sets of genes.

Within the Danish-Swedish Milk Genomics Initiative the milk protein profile of the Danish Holsteins and Danish Jerseys has been studied in detail [6, 7, 9, 24]. Apart from the major genetic variants of the CN genes a study on the genetics underlying the expression of the major milk proteins and their isoform has not been carried out.

The objective of this study was to estimate the heritability of the major milk proteins and their isoforms representing post-translational modifications and to perform a genome-wide association study (GWAS) for the detailed milk protein profile in Danish Holstein (DH) and Danish Jersey (DJ) dairy cattle.

## Methods

### Animals

All samples were taken within the Danish-Swedish Milk Genomics Initiative. “The overall experimental strategy underlying this study was to minimize potential sources of environmental variation and maximize the level of genetic variation in the sample population. As a result, the pedigree of the selected animals was designed to include as unrelated animals as possible“ [25]. In total, the 456 DH cows were sired by 239 bulls and 450 dams, whereas the 436 DJ cows were sired by 152 bulls and 429 dams. Single morning milk samples were collected once from 456 DH cows (20 dairy herds, October - December 2009) to 436 DJ (22 dairy herds, February – April 2010) from conventional herds during the indoor period. Between 19 and 24 cows were sampled from each herd. The cows sampled were all in mid-lactation (d129 to d229 in DH and d130 to d252 in DJ) and within parity 1, 2 or 3. The cows were housed in loose housing systems, fed according to standard practice, and milked twice a day. The milk samples were placed on ice for transport to the laboratory immediately after milking. Once at the laboratory, the milk samples were treated as described by Poulsen et al. [25].

### Milk protein composition

Protein and CN contents were determined in house by infrared spectroscopy (MilkoScan FT2, Foss Electric, Hillerod, Denmark), while SCC was determined by flow cytometry (Fossomatic 5000, Foss Electric, Hillerod, Denmark) at Eurofins Laboratory (Holstebro, Denmark). Samples with SCC >500 × 100 cell/mL were excluded from further study. All milk samples were skimmed by centrifugation for 30 min at 2,643 × *g* at 4 °C. A detailed protein profile (α_S1_-, α_S2_-, β-, and k-CN, β-LG, and α-LA) of the milk was determined in duplicates using liquid chromatography/electrospray ionization-mass spectrometry (LC/ESI-MS) as described in detail by Jensen et al. [6]. Phosphorylation isoforms were identified for α_S1_-CN (8P/9P) and α_S2_-CN (11P/12P) and for k-CN (1P), the respective glycosylated (G-k-CN) and un-glycosylated fractions (U-k-CN) were determined. All proteins and their isoforms were expressed as a percentage of the total protein fraction using the absorbance at 214 nm as basis of integration as described earlier [6]. Furthermore, the glycosylation degree (GD) of k-CN was expressed as G-k-CN/total k-CN, and the phosphorylation degree (PD) of α_S1_-CN and α_S2_-CN was expressed as α_s1_-CN-8P/total α_s1_-CN and α_s2_-CN-11P/total α_s2_-CN, respectively.

### Genotypes and genomic relationship matrix

In total 371 DH and 321 DJ cows were genotyped using the bovine HD SNP array (www.illumina.com/documents/products/datasheets/datasheet_bovineHD.pdf). Genomic DNA was extracted from ear tissue. The platform used was an Illumina® Infinium II Multisample assay device. SNP chips were scanned using iScan and analyzed using Beadstudio version 3.1 software (Illumina, https://www.illumina.com/). The quality parameters used for the selection of SNPs in the GWAS were minimum call rates of 80 % for individuals and 95 % for loci. Marker loci with minor allele frequencies (MAFs) below 1 % were excluded. The quality of the markers was assessed using the GenCall data analysis software of Illumina. Individuals with average GenCall scores below 0.65 were excluded following Teo et al. [26]. The *Bos taurus* genome assembly (*Btau_4.0*) [27] was used to assign the SNP positions on the genome. In total 494,984 SNP markers were used in both DH and DJ. These genotypes were used to calculate a genomic relationship matrix (GRM) as described by VanRaden et al. [28]. In short: **M** is a matrix of n x m specifying which marker alleles each individual inherit, where n = the number of individuals and m = the number of markers. **M** contained elements -1, 0, 1 representing homozygote, heterozygote and the other homozygote, respectively. The diagonals of **M’M** counts the number of homozygous loci for each individual and off diagonals measure the number of alleles shared by relatives. **P** contain the allele frequencies (p_i_), such that column *i* of **P** equals 2(*p*_*i*_-0.5). The allele frequency is then: ${p}_i = \frac{1}{2}\left(\overline{P}+1\right)$. To set the expected mean value to 0, **Z** was created by subtracting **P** from **M**. The genomic relationship matrix **G** was then calculated as **ZZ′**/[2∑p_i_(1-p_i_)] [28].

### Estimation of heritability

Variance components were estimated using the REML approach in DMU [29]. Within each breed, the following model was used in the analysis:

1 $$ {\mathrm{Y}}_{\mathrm{i}\mathrm{jkl}} = \upmu + \mathrm{her}{\mathrm{d}}_{\mathrm{i}} + \mathrm{parit}{\mathrm{y}}_{\mathrm{j}} + {\mathrm{b}}_1 \times \mathrm{D}\mathrm{I}{\mathrm{M}}_{\mathrm{k}} + \mathrm{anima}{\mathrm{l}}_{\mathrm{l}} + {\mathrm{e}}_{\mathrm{i}\mathrm{jkl}} $$

Where Y_ijkl_ is the phenotype of individual l in herd i and lactation j, μ is the fixed mean effect, herd_i_ is a fixed effect (i = 1, 2, …, 20 DH; i =1, 2, …, 22 DJ), parity_j_ is a fixed effect (j = 1, 2, 3 DH, j = 1, 2, 3 DJ), b_1_ is the regression coefficient for DIM_k_, DIM_k_ is a covariate of days in milk (d129 to d229 in DH, d130 to d252 in DJ), and animal is the random additive genetic effect based on **G** of animal l [30].

Univariate analyses were performed to estimate the heritability, which was defined as:

2 $$ {\mathrm{h}}^2 = {\upsigma^2}_{\mathrm{a}}/\ \left({\upsigma^2}_{\mathrm{a}} + {\upsigma^2}_{\mathrm{e}}\right) $$

where σ^2^_a_ was the additive genetic variation, and σ^2^_e_ was the residual variation.

### Association mapping

The association analysis was performed using model 1 extended with an extra covariate for the SNP:b_2_ is the allele substitution effect, SNP_m_ is a covariate indicating if a SNP is homozygote (0,2) or heterozygote (1). The effect of the SNP was tested by a Wald test with a null hypothesis of H_0_: b = 0. The analyses were performed using REML in the R interface of DMU [28] (available at *http://dmu.agrsci.dk*). Significance thresholds were determined using a false discovery rate (FDR) correction using the R package “qvalue” version 1.34.0 (http://github.com/jdstorey/qvalue) [31]. A FDR of *P* < 0.10 was considered significant.

### Linkage disequilibrium along the genome

Local pairwise LD (*r*^2^) between SNP markers on BTA14 was calculated using haploview [32] (Additional file 1: Figure S1 and Additional file 2: Figure S2). Genome-wise pairwise LD was calculated between the SNP markers within each Mb along the genome using the *r*^2^ as a measure based on the software plink v1.07 [33].

### Meta-analysis of the GWAS results

A meta-analysis combining the DH and DJ populations was performed based on the sample size based method as implemented in METAL [34]. In this method an intermediate Z-score is calculated as:

$$ {Z}_i = {\upphi}^{-1}\ \left({P}_i\kern0.1em /\kern0.1em 2\right)* sign\left({\varDelta}_i\right), $$

where *P*_*i*_ is the *P*-value for study *i*, and *Δ*_*i*_ is the direction of the marker effect for study *i*. The statistics is calculated as:

$$ {w}_i=\kern0.75em \sqrt{N_i}, $$

where *N*_*i*_ is the sample size for study i. The overall Z-score is then calculated as

$$ Z=\frac{{\displaystyle {\sum}_i{Z}_i{w}_i}}{\sqrt{{\displaystyle {\sum}_i{w}_i^2}}}, $$

The overall P-value is then calculated as: *P* = 2ϕ(| − *Z*|).

Significance thresholds were determined using a false discovery rate (FDR) correction using the R package “qvalue” version 1.34.0 (http://github.com/jdstorey/qvalue) [31]. A FDR of *P* < 0.10 was considered significant.

### SNPs assigned to genes

The SNPs on the bovineHD chip were mapped to the Btau4.0 assembly. The data from this download contained 26,352 genes with an Entrez Gene ID. For each gene the location on the bovine genome was determined as 5 Kb before the start position of the first exon to 5 Kb after the end position of the last exon. Hence, the defined gene region includes all introns and parts of the upstream and downstream regions of the gene. When a SNP was located in this region it was assigned to the corresponding gene.

## Results

The descriptive statistics for the protein traits in both DH and DJ are reported in Table 1. These are in line with the results presented by Poulsen et al. [32] on the full data, showing that DH has a lower protein (3.43 %) and CN contents (2.66 %) compared to DJ (4.29 % protein, 3.00 % CN). Further, DH has a higher relative concentration of β-CN (36 %) compared to the DJ (β-CN% 28 %), whereas the protein content of α_S1_-CN, α_S2_-CN, k-CN, β-LG, and α-LA were more similar between the two breeds. Differences between breeds in relation to degree of PTMs were observed, both in terms of phosphorylation and glycosylation. There is a difference in the PD of α_S1_-CN and α_S2_-CN, with the less phosphorylated forms being lower in DH compared with DJ, resulting in lower relative amounts of the 8 and 11 P forms of α_S1_- and α_S2_-CN in DH, respectively. On the other hand, fraction of G-k-CN% was higher in DH compared with DJ, with GD for k-CN in DH was 24 % versus 20 % in DJ.

**Table 1:** Mean values, phenotypic standard deviations and heritabilities for milk protein fractions as well as individual milk proteins and their isoforms in Danish Holstein (*n* = 371) and Danish Jersey (*n* = 321) cows^1^

	Danish Holstein			Danish Jersey		
Trait^2^	Mean	SD	h^2^ (SE)	Mean	SD	h^2^ (SE)
Protein%	3.43^a^	0.26	0.47 (0.19)	4.29^b^	0.32	0.70 (0.21)
Casein%	2.66^a^	0.12	0.43 (0.18)	3.00^b^	0.15	0.52 (0.19)
α_s1_-CN%	0.26^a^	0.03	0 (0.12)	0.27^b^	0.03	0.05 (0.10)
α_s2_-CN%	0.05^a^	0.01	0.14 (0.15)	0.06^b^	0.01	0.13 (0.14)
β-CN%	0.36^a^	0.03	0.05 (0.13)	0.28^b^	0.04	0.29 (0.16)
k-CN%	0.058^a^	0.01	0.77 (0.21)	0.069^b^	0.01	0.29 (0.17)
α-LA%	0.03^a^	0.01	0.40 (0.19)	0.02^b^	0.01	0.44 (0.19)
β-LG%	0.08^a^	0.02	0.58 (0.20)	0.06^b^	0.01	0.16 (0.13)
PTM^3^						
8P-α_s1_-CN%	0.19^a^	0.02	0.01 (0.11)	0.21^b^	0.02	0.41 (0.21)
9P-α_s1_-CN%	0.07^a^	0.01	0.25 (0.18)	0.06^b^	0.01	0.23 (0.19)
11P-α_s2_-CN%	0.03^a^	0.01	0.21 (0.17)	0.04^b^	0.01	0.04 (0.15)
12P-α_s2_-CN%	0.019^a^	0.01	0.25 (0.15)	0.018^b^	0.005	0.19 (0.18))
G-k-CN%	0.014^a^	0.004	0.64 (0.20)	0.014^a^	0.004	0.14 (0.18)
U-k-CN%	0.044^a^	0.01	0.71 (0.20)	0.055^b^	0.01	0.27 (0.20)
Indices^4^						
8P-α_s1_-CN/total α_s1_-CN^2^	0.73^a^	0.04	0.34 (0.19)	0.78^b^	0.05	0.56 (0.22)
11P-α_s2_-CN/total α_s2_-CN^2^	0.61^a^	0.06	0.54 (0.20)	0.67^b^	0.05	0.33 (0.19)
G-k-CN/total k-CN^2^	0.24^a^	0.06	0.50 (0.19)	0.20^b^	0.05	0.09 (0.15)

^1^ Details of the full data-set are presented in Poulsen et al. [32]

^2^ Protein and casein (CN) are expressed as percentage traits (g/100 g milk); α_S1_-CN, 8P-α_S1_-CN, 9P-α_S1_-CN, α_S2_-CN, 11P-α_S1_-CN, 12P-α_S2_-CN, β-CN, k-CN, G-k-CN, U-k-CN, α-lactalbumin and β-lactoglobulin are expressed as % of the total protein

^3^ PTM: post translational modification

^4^ Total α_S1_-CN comprises 8P-α_S1_-CN and 9P-α_S1_-CN; Total α_S2_-CN comprises 11P-α_S2_-CN and 12P-α_S1_-CN; Total k-CN comprises G-k-CN 1P and U-k-CN 1P

^a-b^ Mean with different superscript represent a significant difference in the mean (*P* < 0.05) between the Danish Holstein and Danish Jersey

### Heritability

The heritability estimates for the protein traits for both DH and DJ are presented in Table 1. For DH, high heritability estimates were found for protein percentage (0.47), CN percentage (0.43), k-CN (0.77), β-LG (0.58), and α-LA (0.40). For DJ, high heritability estimates were found for protein percentage (0.70), CN percentage (0.52), and α-LA (0.44). With regard to isoforms of specific proteins, DH showed a much higher heritability for 11P-α_S2_-CN, G-k-CN and U-k-CN compared to DJ, whereas the heritability for 8P-α_S1_-CN is much lower in the DH compared to the DJ. This is also reflected in the PD for α_S1_-CN and GD of k-CN (Table 1).

### GWAS

The GWAS results for DH are presented in Additional file 3: Table S1 and for DJ in Additional file 4: Table S2 including the allele-substitution effect, location and annotation.

#### Danish Holstein

In total 11,052 SNP markers have been detected at the FDR <10 % level for protein percentage (200), CN percentage (193), α_S2_-CN% (200), β-CN% (2), k-CN% (4,742), β-LG% (166), G-k-CN% (2,759), U-k-CN% (2,544), 11P-α_S2_-CN% (244), and 12P-α_S2_-CN% (2). No significant SNP markers were detected for α_S1_-CN%, 8P-α_S1_-CN%, 9P-α_S1_-CN% and α-LA% as well as for PD of α_S1_-CN% and α_S2_-CN% and GD for k-CN.

#### Protein percentage versus casein percentage

For protein percentage SNP markers were detected on chromosomes 2, 3, 4, 5, 6, 8, 9, 10, 12, 13, 14, 15, 16, 20, 28 and 29, whereas for CN percentage SNP markers were detected on chromosomes 3, 5, 8, 9, 10, 12, 13, 14, 15, 16, 20, 21 and 23. The SNPs located on BTA6 for protein percentage were located around 44.4 Mb, which is more than 40 Mb apart from the casein cluster on BTA6. No significant markers were detected for CN percentage on BTA6. A comparison between the SNP markers detected for protein percentage and CN percentage revealed that 120 markers were overlapping between these traits with the majority of the overlapping markers located on BTA14 in the DGAT region. The most significant markers for both protein percentage and CN percentage on BTA14 were BOVINEHD1400000275 (rs133271979), and BOVINEHD1400000281 (rs137203218). These two markers are located in the same haplotype block, but are located in a different haplotype block than DGAT (Additional file 1: Figure S1).

#### k casein

Most significant SNPs were detected for k-CN% (4,742). Of these 4,742 SNP markers 2,609 SNP markers were located on BTA6. The most significant quantitative trait locus (QTL) was detected on BTA6 in a region from 87,385,233 bp to 87,421,141 bp spanning the *CSN3* gene. The most significant SNP were BOVINEHD0600023914 (rs110516603) and BOVINEHD0600023927 (rs136864341) with each a –log_10_(*P*-value) = 49.21 explaining 2.7 % of the total variation.

**Fig. 1:**
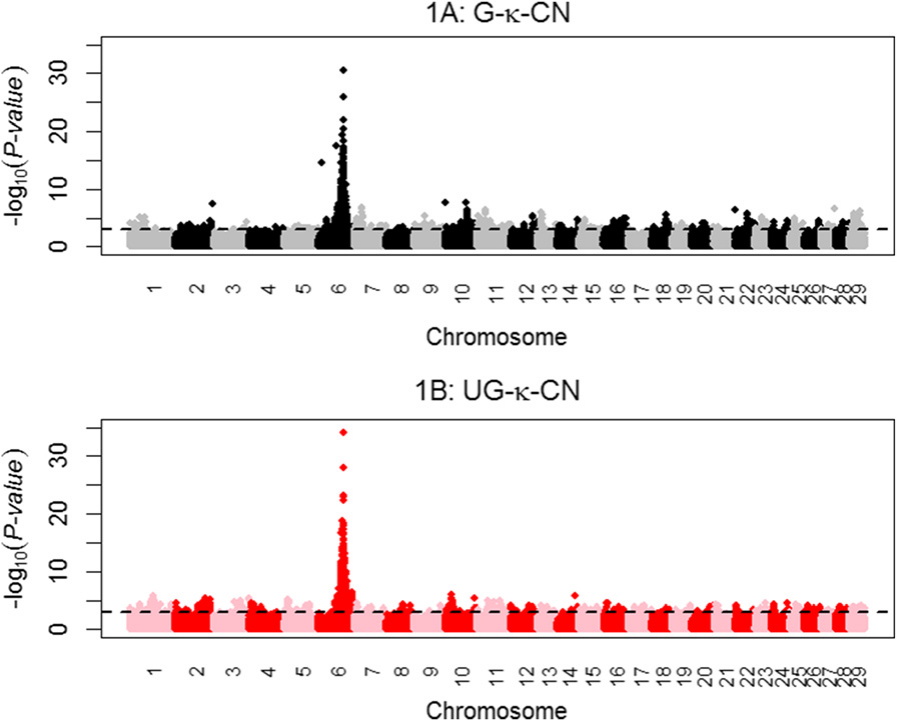
Manhattan plot for G-k-CN (black and grey closed dots) and U- k-CN (red and pink open dots) in Danish Holstein. On the x-axis the chromosomes are represented. On the y-axis the –log_10_ (*P-value*) is presented

The k-CN% is a combination of the glycosylated and un-glycosylated fraction. For the G-k-CN% a total of 2,758 significant SNP markers were detected. Most significant SNP markers were detected on BTA1 (81), BTA6 (1,609), BTA7 (107), BTA10 (125), BTA11 (143), BTA13 (78), and BTA29 (116), whereas for the U-k-CN% a total of 2,544 significant SNP markers were detected. Most significant SNP markers were detected on BTA1 (161), BTA2 (136), BTA6 (1,642), BTA11 (94). Just like k-CN%, both G-k-CN% and U-k-CN% have the majority of the significant SNP on BTA6 close to the k-CN gene. Figure 1 shows the significant markers along the genome for both G-k-CN% and U-k-CN%. Out of the markers significant at FDR < 0.10, 1,039 markers show overlap between G-k-CN% and U-k-CN%, whereas 1,719 SNP markers were specific for G-k-CN%, and 1,505 SNP markers were specific for U-k-CN%. The most significant specific SNP markers for G-k-CN% were BTB-01653149 (rs42768815), BOVINEHD1000020692 (rs132942592), BOVINEHD1000020694 (rs134567350), BOVINEHD1000020695 (rs136061111) and ARS-BFGL-NGS-40559 (rs110826777) on BTA10 each having a –log_10_(*P*-value) = 7.74 explaining 2.1 % of the total variation. These markers are in complete LD (*r*^2^ = 1).The most significant specific SNP markers for U-k-CN% was BOVINEHD1000005681 (rs110977200) on BTA10 with a –log_10_(*P*-value) = 5.99 explaining 16.9 % of the total variation. The location of the peak for G-k-CN% and U-k-CN% on BTA10 were approximately 55 Mb apart. Even though the significant SNP markers for both G-k-CN% and U-k-CN% were located across all autosomes, trait specific peaks were detected on BTA7, BTA11, BTA13, BTA18, BTA22 and BTA29 for G-k-CN% and on BTA1, BTA2, BTA4 and BTA5 for U-k-CN% (Fig. 1). Comparing the GWAS results of k-CN% to the U-k-CN% and G-k-CN% showed that the peaks for G-k-CN% on BTA7, BTA10, BTA18 and BTA22 were specific for G-k-CN%.

#### α_S2_ casein

For α_s2_-CN% SNP markers were detected on chromosomes 1, 3, 5, 6, 8, 9, 11, 14, 18, 19, 22. The majority of the SNP markers was detected on BTA6 (1 70) spanning the CN cluster. The most significant SNP markers were BOVINEHD0600022660 (rs109331183), BOVINEHD0600022661 (rs136565233), BOVINEHD0600022662 (rs110000224) and BOVINEHD0600022663 (rs137452713) with –log_10_(*P*-value) = 8.51. However 244 significant SNP makers were detected for 11P-α_s2_-CN %. These SNPs were divided over eight chromosomes (BTA3, BTA5, BTA 6, BTA9, BTA11, BTA12, BTA19, and BTA28). The majority of the significant SNP markers (222) were detected on BTA6. In total 20 significant SNP markers with a –log_10_(*P*-value) = 9.56 were detected in the range of 87,194,287 bp to 87,296,185 of which 4 SNP markers (BOVINEHD4100005312 (rs110808655), BOVINEHD4100005314 (rs109565340), BOVINEHD4100005315 (rs110122319), BOVINEHD4100005316 (rs109185641)) were assigned to *CSN1S2*.

#### β-LG

For β-LG%, SNP markers were detected on chromosomes 1, 2, 3, 7, 9, 11, 12, 17, and 20. The majority of the significant SNP markers were detected on BTA11 (130). The most significant markers were BOVINEHD1100030066 (rs110186753), BOVINEHD1100030069 (rs110143060) and BOVINEHD1100030070 (rs109087963) with –log_10_(P-value) = 18.84 located in the range of 103,302,351 bp to 103,308,330 bp. BOVINEHD1100030066 and BOVINEHD1100030069 were assigned to the *PAEP* gene (*LGB*).

#### Danish Jersey

In total 287 SNP markers have been detected at the FDR <10 % level for protein percentage (46), CN percentage (60), α_S2_-CN% (21), k-CN% (21), and β-LG (102). 25 SNP markers were detected for 11P- α_S2_-CN%. Furthermore, PD for α_s1_-CN (8P-α_s1_-CN/total α_s1_-CN) and GD for k-CN (Glyc-k-CN/total k-CN) revealed 11 and 1 SNP markers, respectively. No significant SNP markers were detected for β-CN%, α_S1_-CN %, U-k-CN, G-k-CN, 8P-α_S1_-CN %, 9P-α_S1_-CN%, 12P- α_S2_-CN and α-LA% as well as for PD of α_S2_-CN%.

#### Protein percentage versus casein percentage

For protein percentage SNP markers were detected on chromosomes 2, 4, 12, 14 and 20. For CN percentage SNP markers were detected on chromosomes 4, 6, 14, 16 and 20. A comparison between the significant SNP markers for protein percentage and casein percentage revealed that there were overlapping markers on BTA4 (4), BTA14 (26) and BTA20 (2). The four SNP markers on BTA4 were ARS-BFGL-NGS-21411 (rs110554452), BOVINEHD0400020836 (rs134218776), BOVINEHD0400021104 (rs136496474), and ARS-BFGL-NGS-112329 (rs109846161) spanning a distance of 1.2 Mb. The *r*^2^ between the markers was between 0.026 between ARS-BFGL-NGS-21411 and ARS-BFGL-NGS-112329 to 0.40 between BOVINEHD0400021104 and ARS-BFGL-NGS-112329. The overlapping markers detected on BTA14 were located in the DGAT region. Additional file 2: Figure S2 gives an overview of the LD structure in DGAT region for the DJ population, where the most significant markers were detected.

#### k casein

For k-CN% significant SNP markers were detected on BTA6 in the range of 86,627,280 bp to 87,714,272 bp. The most significant markers were BOVINEHD0600023975 (rs109708618) BOVINEHD0600023978 (rs135203089), BOVINEHD0600023979 (rs135983032), BOVINEHD0600023981 (rs137370056), BOVINEHD0600023982 (rs133024540), and BOVINEHD0600023985 (rs110108928) with a –log_10_(*P*-value) = 7.04.

#### α_S2_ casein

For α_S2_-CN% significant SNP markers were detected on chromosomes 2, 6, and 9. The most significant maker on BTA2 was BOVINEHD0200026786 (rs109649678) with a –log_10_(*P*-value) = 6.16. The most significant maker on BTA6 was B OVINEHD0600023975 (rs109708618) with a –log_10_(*P*-value) = 5.79, which was also detected for k-CN. The most significant marker on BTA9 was BOVINEHD0900030427 (rs134789789) at 103.7 Mb with a –log_10_(*P*-value) = 5.79. Furthermore 25 SNP markers were detected for the PTM of 11P-α_S2_-CN%. These markers were detected on BTA2 (6), BTA6 (13), and BTA9 (6). The most significant SNP marker on BTA6 (BOVINEHD0600023003 rs109168832) had a –log_10_(*P*-value) = 6.04.

#### α_S1_ casein

For the PD (8P-α_S1_-CN/total α_S1_-CN), the most significant SNP markers were detected on BTA12 (9). The most significant SNP markers were BOVINEHD1200019300 (rs41668561), BOVINEHD1200019842 (rs134086955), and BOVINEHD1200019848 (rs135231437) with a –log_10_(*P*-value) = 6.64. The markers are located in the range of 70 to 78 Mb. BOVINEHD1200019842 and BOVINEHD1200019848 are located in an unknown gene (http://www.ensembl.org/Bos_taurus/Gene/Summary?db=otherfeatures;g=100337053;r=12:72006073-72205330;t=XM_003586726.2.

For β-LG SNP markers were detected on chromosomes 7, 11, and 16. The majority of the significant SNP markers were detected on BTA11 (93). The most significant markers were BOVINEHD1100030069 (rs110143060) and BOVINEHD1100030070 (rs109087963) with a –log_10_(*P*-value) = 32.16. BOVINEHD1100030069 was assigned to the *PAEP* gene (*BLG*).

### Linkage Disequilibrium in the Danish Holstein and Danish Jersey data-sets

**Fig. 2:**
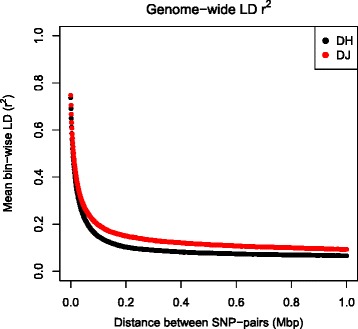
The rate of linkage disequilibrium (LD) for the Danish Holstein (black dots) and Danish Jersey data-set (red dots). On the Y-axis the mean bin-wise LD is presented on the X-axis the physical distance between pair-wise markers is presented in Mega base pairs (Mbp)

The LD analysis of the DH and DJ data-sets showed that there is a difference in the bin-wise LD between these two breeds. The DJ showed higher bin-wise LD across the genome (max mean *r*^2^ = 0.75, min mean *r*^2^ = 0.09) compared to the DH (max mean *r*^2^ = 0.74, min mean *r*^2^ = 0.07) (Fig. 2). The decay with physical distance to reach half of the maximum value (*r*^2^/2 = 0.375) for the DJ was near 23,000 bp, while the decay in DH reaching half of the maximum value (*r*^2^/2 = 0.37) was near 19,000 bp.

### Meta-analysis of the combined Danish Holstein and Danish Jersey data-sets

The results of the meta-analysis in comparison with the within breed analysis are given in Additional file 5: Figure S3, Additional file 6: Figure S4, Additional file 7: Figure S5, Additional file 8: Figure S6 and Additional file 9: Figure S7. No significant markers were detected for α_S1_-CN, 8P-α_S1_-CN, 9P-α_S1_-CN, β-CN, and α-LA as well as for PD of α_S1_-CN% and α_S2_-CN% and GD for k-CN.

#### Protein percentage

The meta-analysis for protein percentage confirmed a major QTL on BTA14 which was detected by both the DH and DJ. In addition a QTL was detected on BTA20 with a strong signal in the meta- analysis. Furthermore a number of smaller meta-analysis peaks show up, but these are mainly due to breed specific signals like BTA2 (in the middle of two QTL peak one specifically for DH and the other for DJ), BTA4 (DJ), BTA12 (DJ) and BTA20 (DJ) (Additional file 5: Figure S3).

#### Casein percentage

BTA14 was confirmed as a major QTL which was detected by both DH and DJ. On BTA25 the meta-analysis QTL peak was much stronger compared to the within breed analysis results. The QTL meta-analysis peaks, which were mainly due to one breed, were detected on BTA4 (DJ), BTA6 (DJ), BTA10 (DH) and BTA20 (DH) (Additional file 6: Figure S4).

#### α_S2_ casein

A major QTL on BTA6 was confirmed by the meta-analysis which was also detected in DH and DJ separately. Furthermore, a QTL on BTA14 was confirmed which was detected by the within breed analysis for both breeds separately. On BTA12, a QTL was detected where the within breed analysis did not show a significant peak. In addition, the meta-analysis showed significant QTL which are mainly due to one breed at BTA2 (DJ), BTA8 (DH), BTA9 (DJ), BTA23 (DJ) (Additional file 7: Figure S5).

#### k casein

The major QTL on BTA6 detected in both breeds was confirmed in the meta-analysis. Furthermore, a few smaller QTL were detected with the meta-analysis on BTA1, BTA5, BTA8 and two QTL peaks on BTA10, BTA11, BTA13, BTA18, two peaks on BTA21, BTA26 and BTA29. The majority of the peaks were due to the DH breed except for BTA5 which was mainly due to DJ. In addition, two QTL peaks were only significant in the DH breed but did not show significance in the meta-analysis (BTA16, and BTA23) (Additional file 8: Figure S6).

#### β-LG

One major QTL was detected on BTA11, which was also revealed as the major QTL in the within breed analysis for both DH and DJ (Additional file 9: Figure S7).

#### Overlapping SNPs between traits

**Fig. 3:**
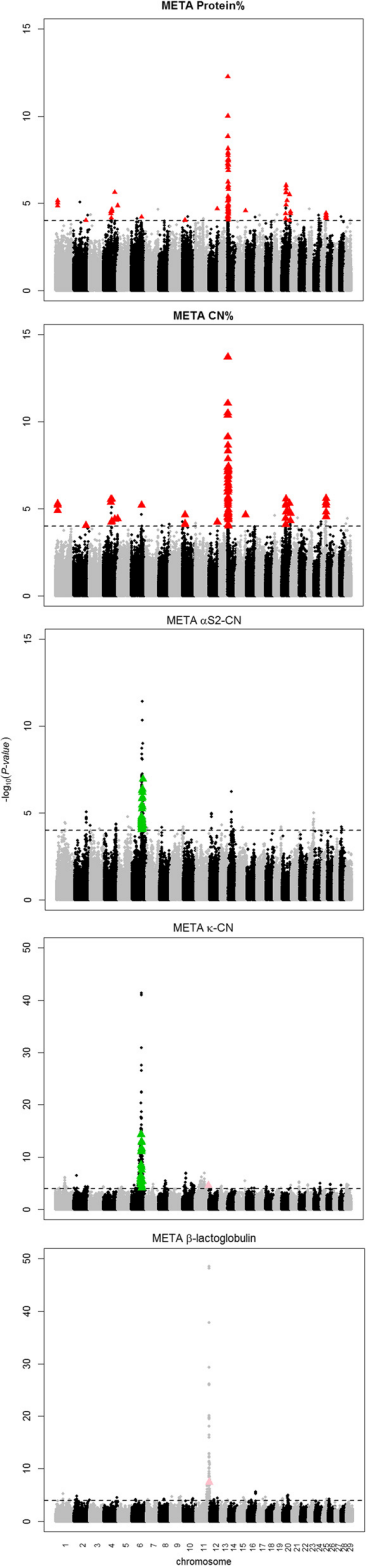
Overview of the distribution of significant markers (FDR < 0.10) of the meta-analysis over the genome for protein% (prot%), casein% (CN%), α_S2_-CN%, k-CN%, β-LG%. Gray dots represent uneven chromosomes, black dots represent even chromosomes. Red triangle: overlapping SNP markers between prot% and CN%, green triangle: overlapping SNP markers between α_S2_-CN%, k-CN%; pink triangle: overlapping SNP markers between k-CN%, β-LG%

Figure 3 shows the distribution of the significant markers from the meta-analysis over the genome and the overlap between the protein traits (protein percentage, casein percentage, α_s2_-CN, k-CN, and β-LG). Relatively low overlap has been found between the individual protein traits. Most overlap was detected for protein percentage and casein percentage on BTA14 (82 SNP markers) followed by BTA20 (15) and BTA25 (14), whereas for α_S2_-CN and k-CN most overlap was detected on BTA6 (147). There was only one SNP marker in common between k-CN and β-LG on BTA11 (BOVINEHD4100009262 (rs109608280)).

## Discussion

This study reports the heritability and GWAS results of the major milk proteins (α_S1_-CN, α_S2_-CN, β-CN, k-CN, β-LG and α-LA), as well as the PTM isoforms; 8P-α_S1_-CN, 9P-α_S1_-CN, 11P- α_S2_-CN, 12P- α_S2_-CN, G-k-CN, U-k-CN, protein and casein contents. Further, GD of k-CN and PD of α_S1_-CN and α_S2_-CN were also explored.

### Heritability

There was a significant difference between the DH and DJ in the protein profile for all traits except for G-k-CN (Table 1) as previously has been shown by Poulsen et al. [24]. The heritabilities of protein and CN percentages were lower in the DH compared to the DJ. In the literature, the heritability for protein percentage covers a wide range from 0.28 [35] up to 0.66 [20]. Such variation in heritability estimates can partly be due to differences in the breeds used, sample size, analytical method, experimental design and statistical models applied. In general the heritability determined in the present study for protein percentage in DJ milk was high compared to values reported in the literature [20, 35, 36], whereas the heritability estimates for the DH were in between the estimated heritabilities presented in the literature [20, 21]. In the DH animals the all 371 animals included in this analysis had genotype BB for the α_S1_-CN gene [8], which could explain the low heritability compared to the studies of e.g. Schopen et al. [20] and Bonfatti et al. [21]. Including more animals and multi-trait analysis could improve the estimation of the heritability of α_S1_-CN% in the DH [37], but it would still be lower compared to values presented in the literature [20, 21].

So far not many studies have focused the genetic analysis of the PTM forms of the milk proteins. Bijl et al. [23] showed differences in heritability for phosphorylation isoforms of α_S1_-CN%. The 8P-α_S1_-CN form had a lower heritability (0.48) compared to the 9P-α_S1_-CN form (0.76) for the Dutch Holstein population. In DH the heritability for 8P-α_S1_-CN is close to 0 most likely for the reason mentioned above. Interestingly, the heritability for 9P-α_S1_-CN is 0.25, which could indicate that other genes than the α_S1_-CN gene can be involved in the formation of 9P-α_S1_-CN. In a recent study the heritability of the glycosylation of k-CN heritability was estimated in Simmental cattle (0.46) [15]. This is a different breed than the DH and DJ, which could give an explanation why the heritability was different (DJ < Simmental < DH). In all three breeds the heritability was substantial indicating that there is room for genetic selection for the glycosylation properties of the milk.

### GWAS of Post Translational Modifications

#### Glycosylation of k-CN

In this study the majority of the QTL for PTM were detected in the DH comparing the results of the G-k-CN versus the U-k-CN (Fig. 1). There was no difference in the mean values of the content of G-k-CN% between DH and DJ, but there was a profound difference in the heritabilities between DH and DJ (heritability DH > DJ (Table 1)). These differences in the heritabilities could potentially explain the difference in the number of QTL detected for G-k-CN and U-k-CN between DH and DJ. The comparison of the GWAS results between k-CN% and the U-k-CN% and G-k-CN% fractions revealed a number of G-k-CN% specific QTL peaks indicating that there is a potential to genetically differentiate between the G-k-CN% and U-k-CN% fraction in the k-CN in the milk. This would be of interest as glycosylation of k-CN is considered to stabilize the micelle structure. A higher fraction of G-k-CN would increase both its charge and the size of the hydrophilic k-CN C-terminal and thereby influence both the cleavage of the k-CN molecule into para k-CN and glycomacropeptide in the first phase of the coagulation process by chymosin as well as the coagulation properties of the milk represented by the second phase [13, 38, 39]. Comparing the results of our study to the study on rheological traits for rennet induced gelation showed that BTA7, BTA10, BTA18 and BTA22 were among the chromosomes identified to play a role in rennet induced coagulation [40]. There was an overlap between the QTL on BTA7 for log(gel strength) and the QTL detected on BTA7 for G-k-CN in our study indicating that QTL for G-k-CN could in part explain the genetic variation of coagulation properties of the milk.

#### Candidate genes

At this stage the information regarding the genetic regulation of the glycosylation of the k-CN is poorly understood. In this study we detected different SNPs which were found to be specific for G-k-CN (Additional file 10: Table S3). The O-glycosylation of k-CN results in glycan attachment at threonine residues resulting in attaching 1 to 6 glycans at specific sites: Thr142, Thr152, Thr154, Thr157 (only variants A and E), Thr163, Thr166, Thr186 [10, 41]. Among the genes assigned to the significant SNP markers there were no obvious candidate genes based on their biological information. It is interesting to mention though that there were two genes which were related to PTMs of caseins. These genes were: Casein kinase 1, Gamma 3 (*CSNK1G3*) on BTA7. This gene is a ubiquitous serine/threonine-specific protein kinase that phosphorylates caseins and other acidic proteins [42], and protein kinase c. theta (*PRKCQ*) on BTA13. Protein kinase C (PKC) is a family of serine- and threonine-specific protein kinases that can be activated by calcium and the second messenger diacylglycerol (http://www.genecards.org/cgi-bin/carddisp.pl?gene=prkcq). However it remains unclear if these genes have a specific role in the glycosylation of k-CN.

#### Phosphorylation of α_S1_-CN and α_S2_-CN

Phosphorylation forms of α_S1_-CN seem to be regulated by a different set of genes [23]. In their GWAS study it was shown that both the 8P and 9P form of α_S1_-CN were regulated by a region on BTA6, while the 8P form was further affected by a region harboring the *PEAP* gene on BTA11, while the 9P form was additionally regulated by a region harboring *DGAT1* on BTA14 [23]. In our study, we identified a region on BTA12 in DH for 8P-α_S1_-CN%. Further we identified regions on BTA6 and BTA11, these were, however, not significant. The reason for this could be that the number of animals used in the GWAS analysis in our study is relatively small compared to the mapping population size of Bijl et al. [23]. A smaller population size results in a lower power to detect an association, and therefore could explain the missing overlap.

### GWAS of major proteins

#### Single GWAS versus meta-analysis GWAS

In this study, within population GWAS was followed by a meta-analysis for the major milk proteins and their PTM forms. As single GWAS are underpowered due to small sample sizes, meta-analysis which combines information from independent studies can increase power and reduces false-positive findings [43]. However the increase in power in the meta-analysis can only be seen when the same loci influencing the trait of interest are segregating in both populations but are not significant in the single population GWAS study due to lack of power. In such cases the meta-analysis could enhance the association signal and detection probability. We studied two distinct dairy cattle breeds (DH and DJ). They differ both phenotypically in the milk composition [8, 44], as well as in their genetic make-up as these breeds have been genetically separated for many generations [45] and have undergone strong artificial selection. This is reflected in the difference in the LD structure of the DH and DJ samples used in this study (Fig. 2). Furthermore, it has been shown that genomic prediction across Holstein and Jersey populations are difficult [46]. This is reflected in the meta-analysis of this study, the majority of the QTL which was considered significant was also significant in either the DH or DJ population except for the QTL on BTA12 for α_S2_-CN% (Additional file 7: Figure S5).

#### Major QTL regions

If the genome-wide Bonferroni correction for multiple testing was applied only three major regions for the protein composition would be detected both in the DH and DJ: BTA6 (k-CN) covering the CN gene complex, BTA14 (protein percentage and casein percentage) covering the *DGAT* gene and BTA11 (β-LG) covering the *PEAP* gene. This is in line with the findings of Schopen et al. [22]. Interestingly when analyzed as a yield trait the QTL on BTA14 for protein was not detected [22]. This is in line with the findings of Bovenhuis et al. [47] who detected significant association with mineral composition in the milk and DGAT when analyzed as a percentage trait, while analyzed as a yield trait the association with DGAT disappeared. This suggested that the QTL on BTA14 has an indirect effect on protein and casein percentage [47].

## Conclusion

The genetic analysis of the major milk proteins and their PTM forms revealed that these were heritable in both the DH and the DJ. Furthermore genomic regions for the major milk proteins confirmed the regions on BTA6 (CN cluster), BTA11 (*PEAP*), and BTA14 (*DGAT1*) as important regions influencing protein composition in the milk. The genetic analysis k-CN and the U-k-CN and G-k-CN showed specific genomic regions regulating the glycosylation of k-CN in the DH. The results from this study provide confidence that it is possible to breed for specific milk protein composition and its molecular forms in the future.

## Abbreviations

α-LA, α-lactoalbumin; β-LG, β-lactoglobulin; CN, casein; DH, Danish Holstein; DJ, Danish Jersey; FDR, False discovery rate; GD, glycosylation degree; GWAS, genome-wide association study; LD, linkage disequilibrium; PD, phosphorylation degree; PTM, post-translational modifications; QTL, quantitative trait loci.

## Additional files

Additional file 1: Figure S1. Linkage disequilibrium plot (r^2^) of the DGAT region in the Danish Holstein breed. (PNG 357 kb) Additional file 2: Figure S2. Linkage disequilibrium plot (*r* ^2^) of the DGAT region in the Danish Jersey breed. (PNG 342 kb) Additional file 3: Table S1. Significant SNP markers (FDR < 0.10) for the major milk proteins and their post translational modified forms for Danish Holstein cattle. (TXT 2300 kb) Additional file 4: Table S2. Significant SNP markers (FDR < 0.10) for the major milk proteins and their post translational modified forms for Danish Jersey cattle. (TXT 58 kb) Additional file 5: Figure S3. Manhattan plot for protein % for the within breed analysis (black and grey closed dots: Danish Holstein; green/blue closed dotes: Danish Jersey) and the meta-analysis (pink and red closed dots). The horizontal dashed line represents FDR < 0.10 significance level. On the x-axis the chromosomes are represented. On the y-axis the –log_10_ (*P-value*) is presented. (PNG 14 kb) Additional file 6: Figure S4. Manhattan plot for casein % for the within breed analysis (black and grey closed dots: Danish Holstein; green/blue closed dotes: Danish Jersey) and the meta-analysis (pink and red closed dots). The horizontal dashed line represents FDR < 0.10 significance level. On the x-axis the chromosomes are represented. On the y-axis the –log_10_ (*P-value*) is presented. (PNG 14 kb) Additional file 7: Figure S5. Manhattan plot for α_S2_-CN% for the within breed analysis (black and grey closed dots: Danish Holstein; green/blue closed dotes: Danish Jersey) and the meta-analysis (pink and red closed dots). The horizontal dashed line represents FDR < 0.10 significance level. On the x-axis the chromosomes are represented. On the y-axis the –log_10_ (*P-value*) is presented. (PNG 14 kb) Additional file 8: Figure S6. Manhattan plot for k-CN% for the within breed analysis (black and grey closed dots: Danish Holstein; green/blue closed dotes: Danish Jersey) and the meta-analysis (pink and red closed dots). The horizontal dashed line represents FDR < 0.10 significance level. On the x-axis the chromosomes are represented. On the y-axis the –log_10_ (*P-value*) is presented. (PNG 10 kb) Additional file 9: Figure S7. Manhattan plot for β-LG% for the within breed analysis (black and grey closed dots: Danish Holstein; green/blue closed dotes: Danish Jersey) and the meta-analysis (pink and red closed dots). The horizontal dashed line represents FDR < 0.10 significance level. On the x-axis the chromosomes are represented. On the y-axis the –log_10_ (*P-value*) is presented. (PNG 11 kb) Additional file 10: Table S3. Significant SNP markers (FDR < 0.10) unique for G-k-CN compared to U-k-CN in Danish Holstein cattle. (TXT 87 kb)
