# Supplementary figures and images for: Estimation of genetic parameters and detection of chromosomal regions affecting the major milk proteins and their post translational modifications in Danish Holstein and Danish Jersey cattle
Source: BMC Genet. 2016 Aug 2;17:114. doi: 10.1186/s12863-016-0421-2 (PMC4969662; doi:10.1186/s12863-016-0421-2)

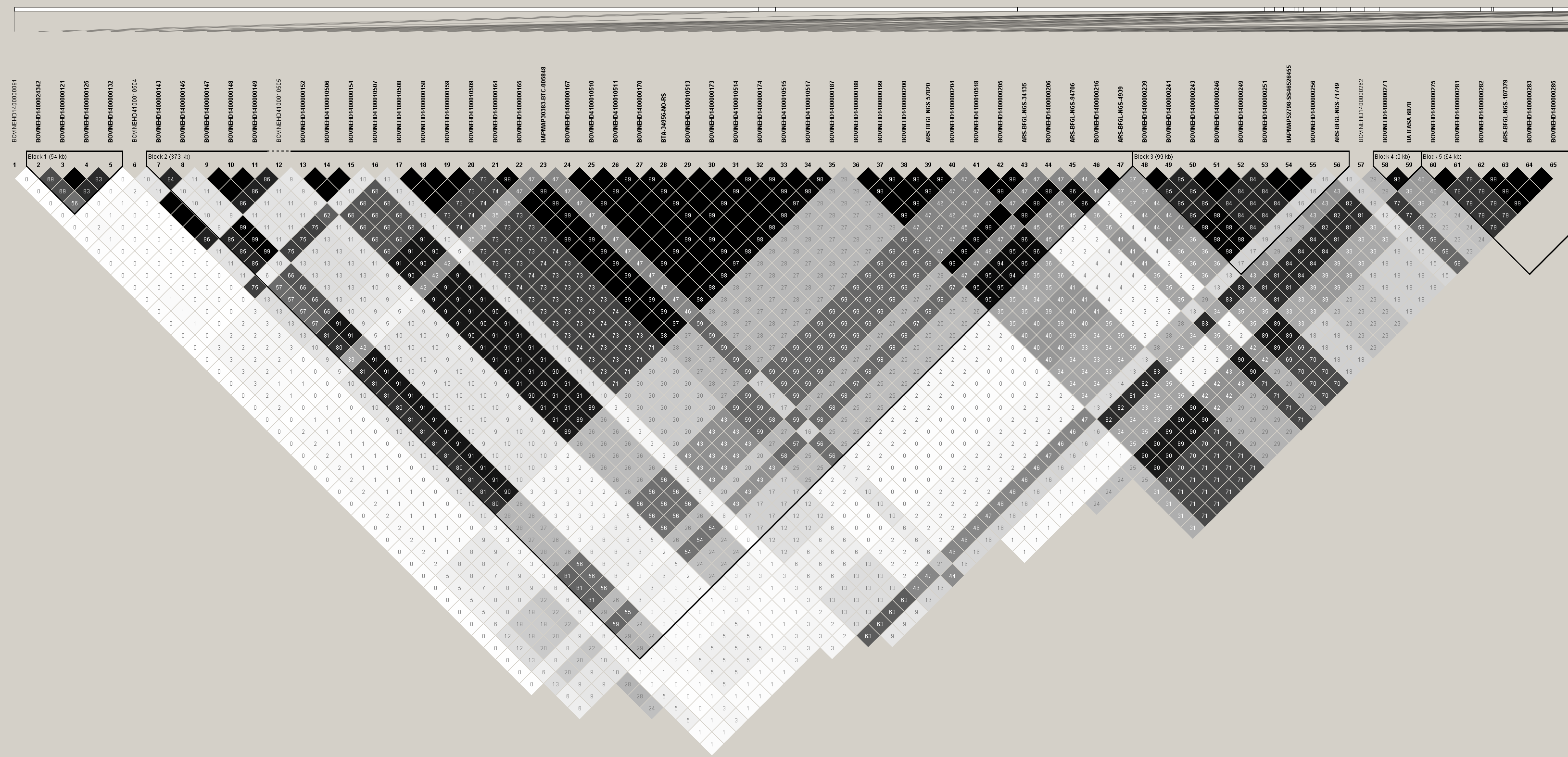

Supplement: Additional file 1: Figure S1. — Linkage disequilibrium plot (r2) of the DGAT region in the Danish Holstein breed. (PNG 357 kb) [file 12863_2016_421_MOESM1_ESM.png]

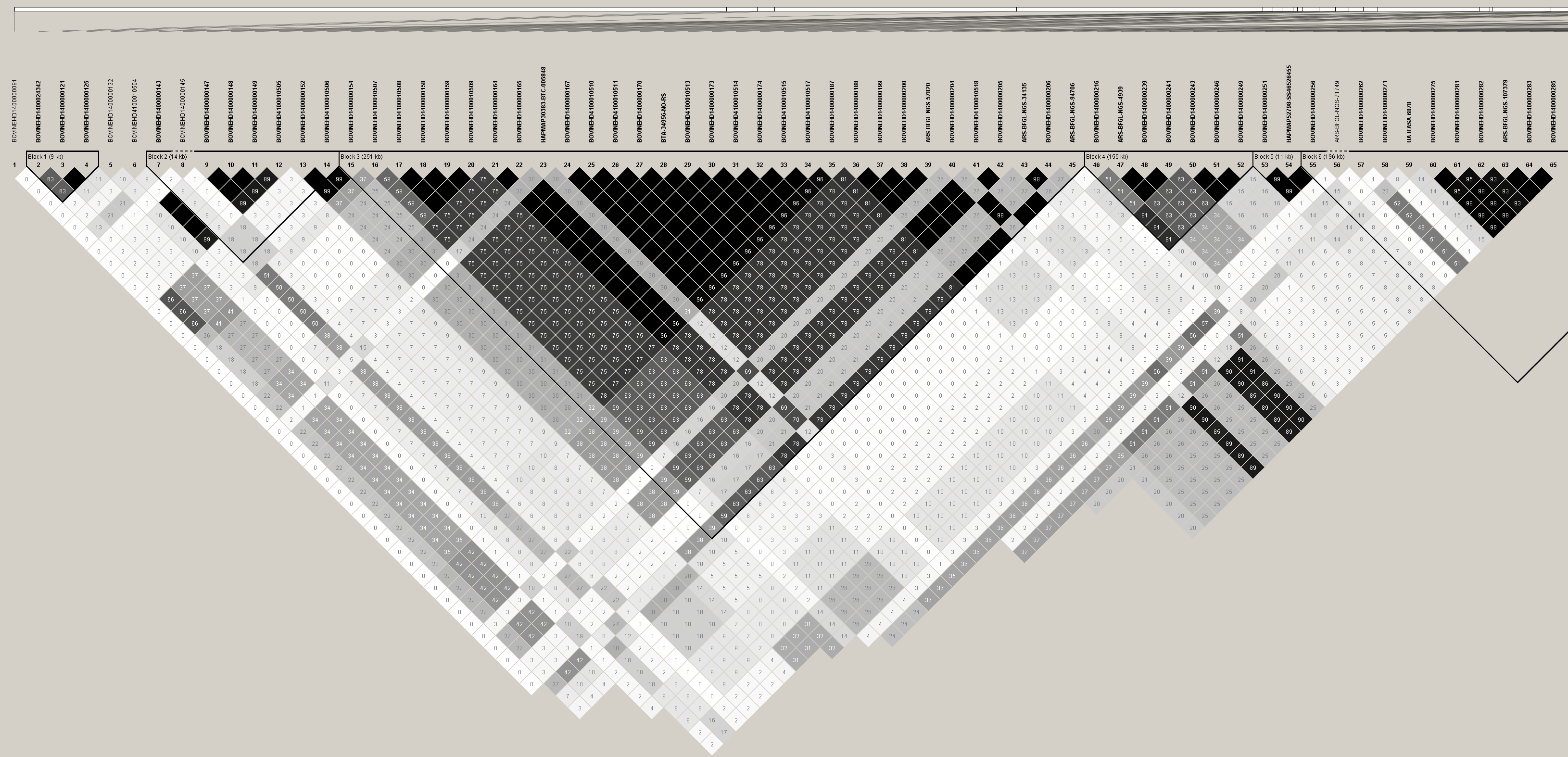

Supplement: Additional file 2: Figure S2. — Linkage disequilibrium plot (r 2) of the DGAT region in the Danish Jersey breed. (PNG 342 kb) [file 12863_2016_421_MOESM2_ESM.png]

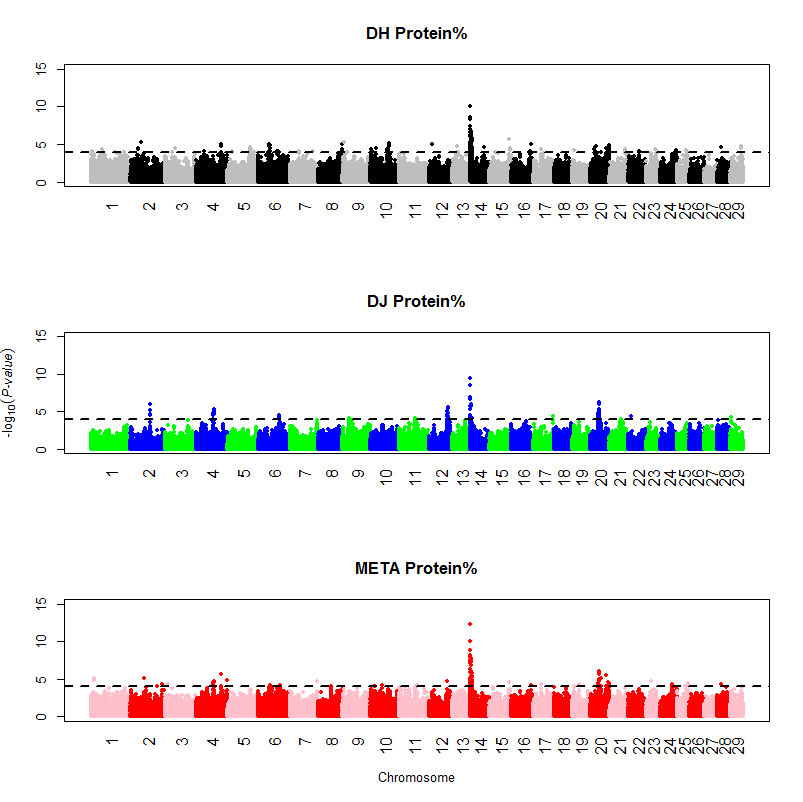

Supplement: Additional file 5: Figure S3. — Manhattan plot for protein % for the within breed analysis (black and grey closed dots: Danish Holstein; green/blue closed dotes: Danish Jersey) and the meta-analysis (pink and red closed dots). The horizontal dashed line represents FDR < 0.10 significance level. On the x-axis the chromosomes are represented. On the y-axis the –log10 (P-value) is presented. (PNG 14 kb) [file 12863_2016_421_MOESM5_ESM.png]

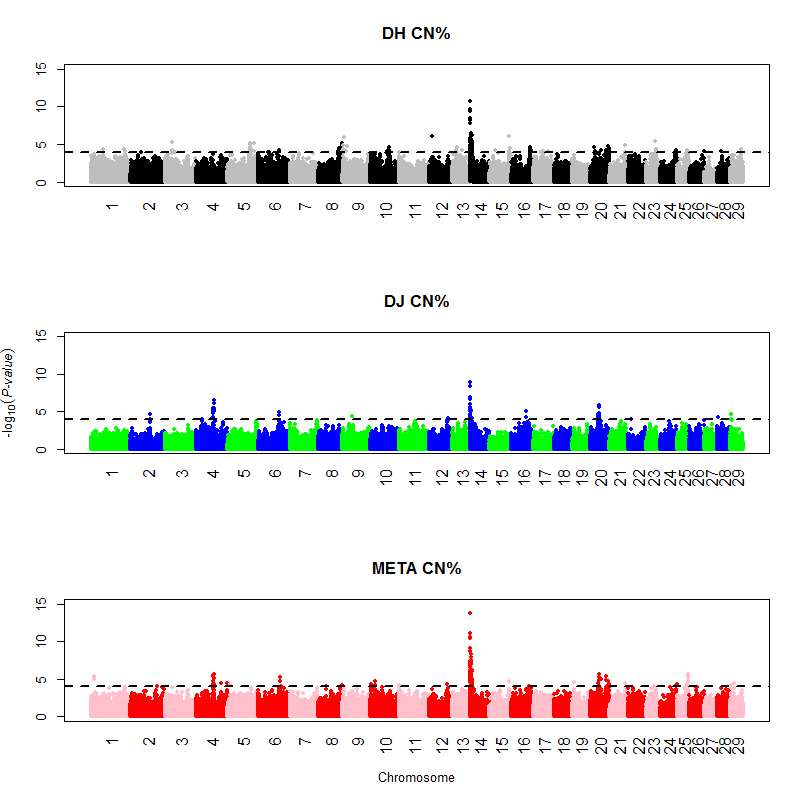

Supplement: Additional file 6: Figure S4. — Manhattan plot for casein % for the within breed analysis (black and grey closed dots: Danish Holstein; green/blue closed dotes: Danish Jersey) and the meta-analysis (pink and red closed dots). The horizontal dashed line represents FDR < 0.10 significance level. On the x-axis the chromosomes are represented. On the y-axis the –log10 (P-value) is presented. (PNG 14 kb) [file 12863_2016_421_MOESM6_ESM.png]

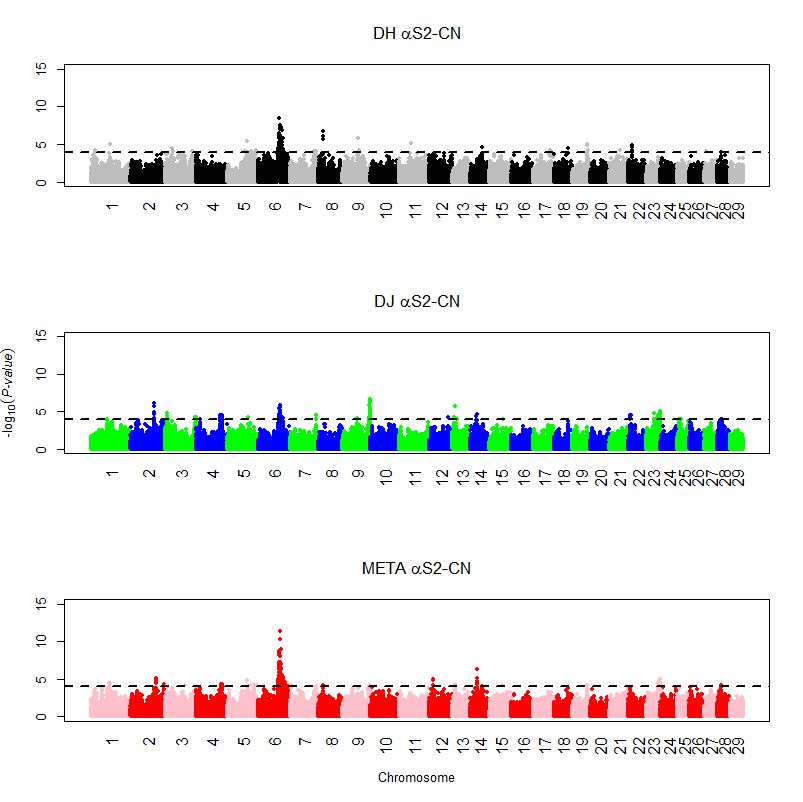

Supplement: Additional file 7: Figure S5. — Manhattan plot for αS2-CN% for the within breed analysis (black and grey closed dots: Danish Holstein; green/blue closed dotes: Danish Jersey) and the meta-analysis (pink and red closed dots). The horizontal dashed line represents FDR < 0.10 significance level. On the x-axis the chromosomes are represented. On the y-axis the –log10 (P-value) is presented. (PNG 14 kb) [file 12863_2016_421_MOESM7_ESM.png]

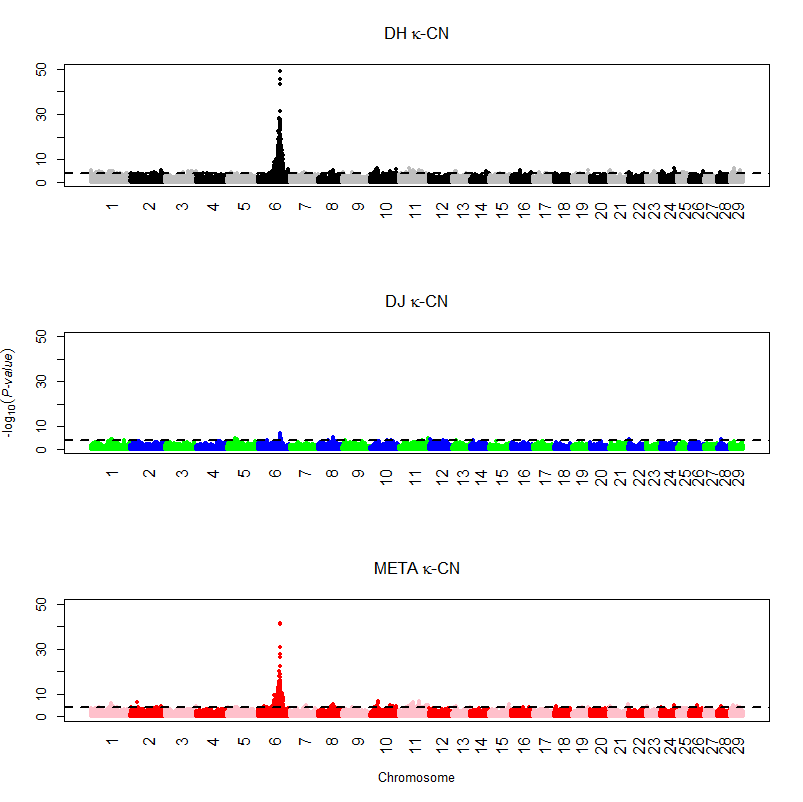

Supplement: Additional file 8: Figure S6. — Manhattan plot for k-CN% for the within breed analysis (black and grey closed dots: Danish Holstein; green/blue closed dotes: Danish Jersey) and the meta-analysis (pink and red closed dots). The horizontal dashed line represents FDR < 0.10 significance level. On the x-axis the chromosomes are represented. On the y-axis the –log10 (P-value) is presented. (PNG 10 kb) [file 12863_2016_421_MOESM8_ESM.png]

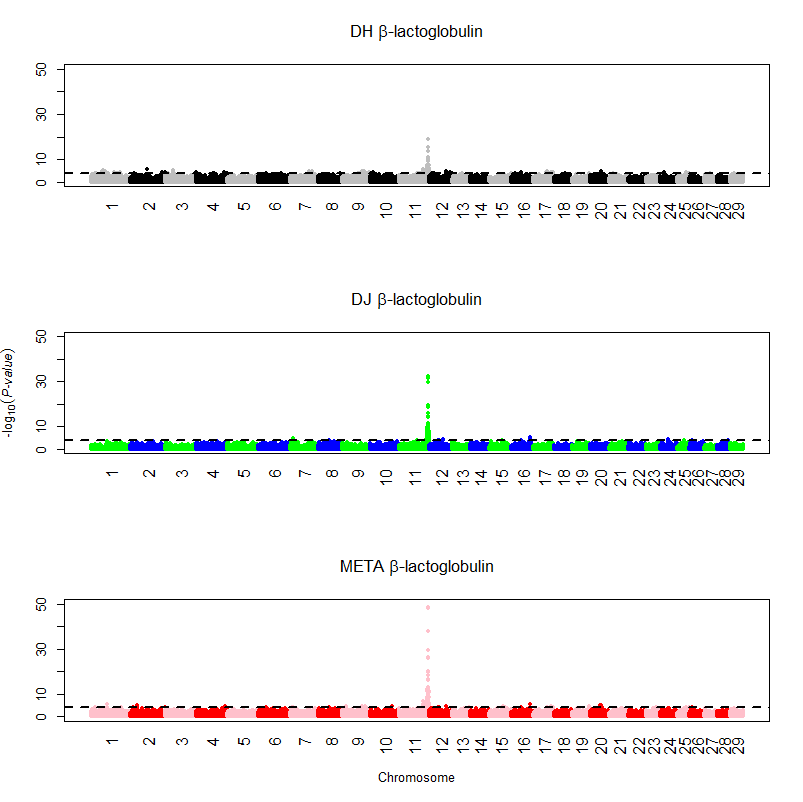

Supplement: Additional file 9: Figure S7. — Manhattan plot for β-LG% for the within breed analysis (black and grey closed dots: Danish Holstein; green/blue closed dotes: Danish Jersey) and the meta-analysis (pink and red closed dots). The horizontal dashed line represents FDR < 0.10 significance level. On the x-axis the chromosomes are represented. On the y-axis the –log10 (P-value) is presented. (PNG 11 kb) [file 12863_2016_421_MOESM9_ESM.png]
